# High Mobility Group Box 1 Protein Induction by *Mycobacterium Bovis* BCG

**DOI:** 10.1155/2007/53805

**Published:** 2007-11-18

**Authors:** Péter Hofner, György Seprényi, András Miczák, Krisztina Buzás, Zsófia Gyulai, Katalin F. Medzihradszky, Ari Rouhiainen, Heikki Rauvala, Yvette Mándi

**Affiliations:** ^1^Department of Medical Microbiology and Immunobiology, University of Szeged, Szeged 6720, Hungary; ^2^Department of Medical Biology, University of Szeged, Szeged 6720, Hungary; ^3^Proteomics Research Group, Biological Research Center of the Hungarian Academy of Sciences, Szeged 6720, Hungary; ^4^Department of Pharmaceutical Chemistry, University of California, San Francisco, CA 94143, USA; ^5^Laboratory of Molecular Neurobiology, University of Helsinki, Helsinki 00014, Finland

## Abstract

High mobility group box 1 protein (HMGB1), a nuclear protein, is a critical cytokine that mediates the response to infection, injury, and inflammation. The aim of our study was to elaborate a reliable in vitro model to investigate whether *Mycobacterium bovis* BCG is able to induce HMGB1 secretion from the monocytic U-937 cells. Western blot technique was applied for the detection of HMGB1 from supernatants of cells, following induction with *Mycobacterium bovis* BCG. Densitometric analysis revealed higher concentrations of HMGB1 in cell supernatants stimulated with BCG than in the supernatants of the control, nonstimulated cells. Further quantitation of the secreted HMGB1 was performed by ELISA. The BCG strain resulted in a higher amount of secreted HMGB1 (450 ± 44 ng/mL) than that of LPS (84 ± 12 ng/mL) or *Staphylococcus aureus* (150 ± 14 ng/mL). BCG and Phorbol −12-myristate −13 acetate (PMA), added together, resulted in the highest HMGB1 secretion (645 ± 125 ng/mL). The translocation of the HMGB1 towards the cytoplasm following infection of cells with BCG was demonstrated by immunofluorescence examinations. Conclusion: Our pilot experiments draw attention to the HMGB1 inducing ability of *Mycobacterium bovis*. Assesment of the pathophysiological role of this late cytokine in mycobacterial infections demands further in vitro and in vivo examinations.

## 1. INTRODUCTION

High mobility group box chromosomal protein 1 (HMGB1) is an ubiquitous, extremely conserved (99% mammal identity) protein, to have DNA-binding properties; to participate in DNA transcription, replication, and repair; and also to participate in neurite outgrowth and cell motility [1, 2]. Amphoterin is a 30-kD heparin-binding protein widely expressed in humans and other organisms, and it is abundantly expressed in the developing brain as well as in various immature and transformed cell lines. Unexpectedly, its amino acid sequence turned out to be identical to HMGB1 [3]. In a new nomenclature of high-mobility group proteins, amphoterin is called HMGB1 [4]. Further investigations revealed that this has properties similar to those of proinflammatory cytokines [5–8]. HMGB1 has been demonstrated to have the capacity to induce cytokines and to activate inflammatory cells when it is released extracellularly [8–10]. Extracellular HMGB1 functions as a proinflammatory late cytokine, and activates monocytes and endothelial cells, leading to release of further cytokines [11, 12]. To act as a danger signal and inflammatory mediator, HMGB1 must be transported extracellularly [13]. Moreover, HMGB1 can also be actively secreted by stimulated macrophages or monocytes in a process requiring acetylation of the molecule, which enables translocation from the nucleus to secretory lysosomes [14]. In response to proinflammatory stimuli, that is, proinflammatory cytokines such as TNF-α and IL-1, or even. lipopolysacharide (LPS), HMGB1 may be actively secreted from monocyres and macrophages via the nonclassical, vesicle-mediated pathway [15–17]. At the same time, HMGB1 is a nuclear danger signal passively released by necrotic cells that will induce inflammation [18], but not from cells undergoing apoptosis.

In the classical experiment of Wang et al. [19] when LPS was administered to mice to generate endotoxaemia, the serum HMGB1 levels began to increase 12–18 hours after the peak levels of TNF, and IL-1. Administration of HMGB1 to mice causes sepsis-like symptoms and death. HMGB1 has been found as a downstream and late mediator in human sepsisl too [20, 21]. During infections, microbial components can be the activators of danger signals. So in infectious diseases, HMGB1 may function as a secreted or released molecule to alert the host that the pathogen has invaded. Gram-negative and Gram-positive bacteria are able to cause sepsis, but little is known about the HMGB1 inducing ability of mycobacteria. We therefore set out to elaborate an in vitro model, with which the HMGB1 inducing capacities of different bacteria or bacterial components could be investigated. For this purpose, the monocytic cell line U-937 were applied, and the extents of HMGB1 secretion were compared following activation of these cells with *E. coli* LPS, *Staphylococcus aureus*, and *Mycobacterium bovis* BCG.

## 2. MATERIALS AND METHODS

### 2.1. Cell line

The human monocytic cells U-937 were propagated in RPMI 1640 medium supplemented with 100 *μ*g/mL ampicillin, 100 *μ*g/mL streptomycin, and 10% heat-inactivated fetal calf serum (FCS, GIBCO) at 37°C in a humidified CO_2_ incubator.

### 2.2. Cell stimulation

To analyze HMGB1 secretion from U-937 cells, culture media were replaced by OPTIMEM (GIBCO) medium, and the cell number was adjusted to 10^6^ cells/mL. The cells were stimulated for 24 hours with: 5 ng/mL Phorbol −12-myristate −13 acetate (PMA, SIGMA), 10 *μ*g/mL LPS *E. coli* O111: B4 (SIGMA), heat-killed *Staphylococcus aureus (SA)* 10^8^/mL, or with *Mycobacterium bovis BCG* (10^7^/mL).

### 2.3. Mycobacterial strain and growth conditions

The Pasteur strain of *M. bovis* BCG (bacille Calmette-Guèrin) was kindly provided by David G. Russel (Department of Microbiology and Immunology, Cormell University, Ithaca, NY, USA). Bacteria were grown at 37°C in Middlebrook broth (Difco Laboratories, Detroit, Mich, USA) with OADC supplement (oleic acid, albumin fraction V. dextrose and catalase) containing 0.05% Tween-80. The chemicals used to prepare the OADC supplement were purchased from Sigma (DC, USA). Heat-killed bacteria were treated at 85°C for 30 minutes.

### 2.4. HMGB1 western blot analysis

Supernatants of activated U-937 cells were first concentrated on a Centricon 10, with subsequent processing in Laemmli buffer [22]. The 10-fold concentrated supernatants and HMGB1 standards (human recombinant HMGB1; SIGMA; and recombinant rat amphoterin-recAtn [23, 24]) were resolved in 12.5% SDS-PAGE under reducing conditions. After electrophoresis, proteins were transferred to nitrocellulose blotting membrane (Trans Blot BioRad) using a semidry HOEFER transfer system. Membranes were blocked overnight with 5% nonfat dry milk in PBS containing 0.05% Tween. Filters were stained with affinity-purified polyclonal chicken antihuman HMGB1 antibody [3, 23], followed by a horseradish peroxidase-conjugated goat antichicken antibody (ZYMED Laboratories, San Francisco, Calif, USA) and developed with ECL-Plus (Amersham Pharmacia), and then followed by exposure to X-ray film (KODAK BIOMAX). Densitometric analysis of the blots was performed by Image Quant software (Amersham Bioscience , Buckinghamshire, UK).

### 2.5. Mass spectrometry

*In-gel digestion*: 1D SDS-PAGE separated, silver- or Coomassie Brilliant Blue-stained protein bands were cut, diced, and washed with 25 mM NH_4_HCO_3_ in 50% (v/v) acetonitrile/water. After reduction with dithiothreitol (Sigma, 30 min, 56°C) and alkylation with iodoacetamide (30 min, room temperature, in the dark) the proteins were digested in the gel with side-chain-protected, porcine trypsin (Promega, Madison, Wis, USA.) in 25 mM NH_4_HCO_3_ buffer (pH *∼* 7.8) for 4 hours at 37°C. Tryptic digests were extracted and desalted on C18 ZipTip (Millipore, Bedford, Mass, USA.).

MS analysis of the unfractionated tryptic digests was performed in positive-ion, reflectron mode, on a Reflex III MALDI-TOF (matrix-assisted laser-desorption ionization time-of-flight) mass spectrometer (Bruker, Karlsruhe, Germany), using 2.5-dihydroxybenzoic acid as the matrix. Two-point external calibration was applied, which guarantees a mass accuracy within 200 ppm.

The peak lists were generated with X-Tof (version 5.1.5) software. Masses detected were submitted to a database search on the NCBInr database (NCBInr 2005.10.29. 2972605 sequences; 1023918613 residues). The protein identification was confirmed by sequence information obtained from MS/MS (postsource decay) spectra, collected in 10–12 steps, lowering the reflector voltage by 25% in each step, and then stitching the data together.

Low-energy CID experiments were performed on an Agilent Technologies XCT Plus (Agilent Technologies, Santa Clara, Calif, USA) equipped with an AP MALDI source. For these experiments 4-hydroxy-α cyano-cinnamic acid was used as the matrix.

**HMGB1 ELISA**

The HMGB1 concentrations were determined with a recently established available ELISA kit (Shino-Test Corp, Shagamihara, Japan) [24]. Briefly, in the wells coated with anticalf HMGB1 monoclonal antibody, samples to be measured and standards—*E. coli*-derived HMGB1 (SIGMA)—were incubated for 24 hours at 37°C. After washing the wells for 5 times, a peroxidase-conjugated anti-HMGB1 monoclonal antibody was added into the microwells and incubated for 120 minutes at room temperature. After washing the wells for 5 times, 100 uL of substrate solution was added and incubated for 30 minutes at roome temperature. A stop solution was added, and the absorbance was measured at 450 nm (650 nm reference) using microplate reader (Anthos Labtech Instruments Eugendorf, Austria). The concentrations of the samples were then calculated from the standard curve.

**TNF ELISA**

The TNF-α concentrations in the supernatants of the control and stimulated cells were quantified by using TNF-α ELISA kits (BIOSOURCE), according to the instructions of the manufacturer.

### 2.6. Immunofluorescence

Nonstimulated (control) or activated U-937 cells, cultured on glass coverslips, were fixed in 4% paraformaldehyde for 15 minutes at room temperature. Thereafter the cells were washed 3 times with PBS and permeabilized with 0.3% Triton X-100 in PBS for 10 minutes at room temperature. After 3 washing with PBS, the coverslips were saturated with 10% BSA in PBS for 1 hour. Cells were stained with chicken anti-rec Atn antibody [3, 23] for 1 hour. Bound antibody was detected with FITC-conjugated donkey antichicken IgY (Jackson Immunoresearch Laboratories, Baltimore, Pike, Pa, USA) for 45 minutes. Between all incubation step, the cells were washed three times with PBS containing 0.2% BSA. Coverslips were mounted on slides using Moviol (SIGMA). Fluorescence signals were analyzed by confocal microscopy.

### 2.7. Confocal microscopy and semiquantitative assessment of fluorescence intensities

Serial images of the immunostained samples were captured by Olympus FV1000 confocal laser scanning microscope with standard parameter settings.

The immunofluorescence of control, BCG and PMA treated cells was quantitatively analyzed by ImageQuant software (Molecular Dynamics) as follows: 30 equal circular areas covering the cells were randomly selected on each image; the backgrounds of the selected areas were eliminated by threshold set up; and the fluorescence intensities/pixel values of the randomly selected cells were quantified.

The mean and the standard deviation of the data gained from serial circular areas were calculated by the Microsoft Excel. The level of statistical significance was determined by two-tailed t-probe.

### 2.8. Statistical analysis

All values are expressed as means ± standard deviation (SD). Sudent’s paired t-test was used for comparisons, and *P* values of less than .05 were considered statistically significant.

## 3. RESULTS

### 3.1. Identification of HMGB1 by western blotting

**Figure 1:**
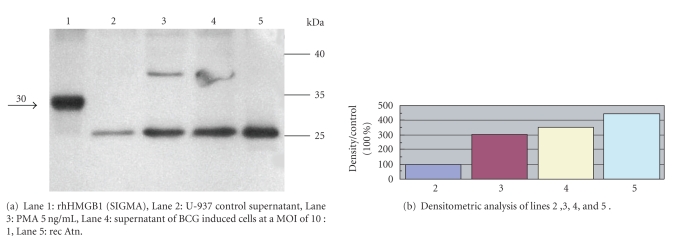
Western blot analysis of U-937 supernatants for HMGB1. Supernatants 10-fold concentrated by Centricon 10, and HMGB1 standards (100 ng) were run under reducing conditions on the gel (12.5% SDS-PAGE), transferred to nitrocellulose. Filters were stained with affinity-purified polyclonal chicken anti- HMGB1 antibody, followed by a horseradish peroxidase-conjugated goat antichicken antibody (ZYMED), developed with with ECL-Plus (Amersham, Pharmacia) followed by exposure to X-ray film (KODAK BIOMAX). Densitometric analysis of the blots were performed by Image Quant software (Amersham Bioscience).

Western blotting of concentrated supernatants with affinity purified antirec Atn antibodies revealed a 25 kD band which comigrated with recAtn. *E. coli* derived recombinant human HMGB1 gave also a considerable signal with this antibody, but the two proteins featured different molecular weights; rec Atn was detected at 25 kD, similarly to the samples from U-937 cell supernatants. (Figure 1), and *E. coli* derived recombinant human HMGB1 at 30 kD. In these experiments affinity-purified chicken IgY antibody against recombinant rat Amphoterin (recAtn) was used [3], because the secreted form of HMGB1 from cell supernatants was recognized by this antibody satisfactory. Commercial monoclonal antibodies failed to form considerable bands in Western blot experiments (personal observations). The chicken antibody was raised against recombinant rat Amphoterin (rec Atn), but the rat amphoterin/HMGB1 differs only with respect to two amino acids from the human protein (Asp189 Glu and Glu 201 Ap) [3].

The level of HMGB1 was assessed by Western blotting and by measuring the intensities of the immunoreactive bands at 25 kD. Densitometric analysis revealed higher concentrations of HMGB1 in cell supernatants stimulated either with phorbol esther, or BCG (Figure 1) than in the supernatants of the control, nonstimulated cells.

### 3.2. Identification of HMGB1 by mass spectometry

As in Western blot experiments, two different positive controls (i.e., *E. coli* derived human recombinant HMGB1 and rec Atn) were used, and the two proteins featured different molecular weights (30 kD and 25 kD, resp.), a mass spectometry analysis was performed for the identification of protein.

**Figure 2:**
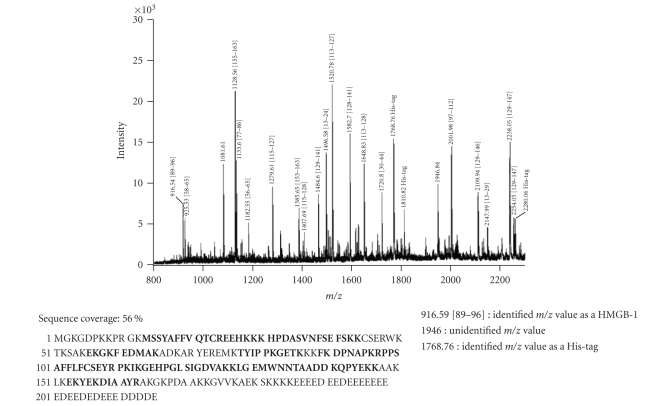
Mass spectral identification of recombinant human HMGB-1 from *E. coli.* Database search with MS data identified the 30 kDa band as HMGB-1 protein, which was confirmed by MS/MS spectra of m/z 1128.56 matching the sequence YEKDIAAYR of HMGB-1 and 1768.76 matching the sequence GSSHHHHHHSSGLVPR of hexahistidine peptide (His-tag). 79% of the masses detected matched this protein. Sequence positions are in square brackets.

**Figure 3:**
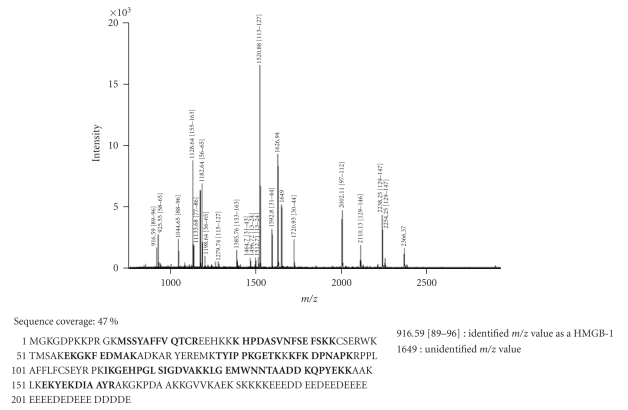
Mass spectral identification of recombinant rat HMGB1. Database search of the PMF data identified the 25 kDa band as HMGB-1 protein, which was confirmed by a PSD spectrum of m/z 1520.78 matching the sequence IKGEHPGLSIGDVAK of HMGB-1. 82% of the masses detected matched this protein. Sequence positions are in square brackets.

The identity of both proteins was confirmed unambiguously. In-gel tryptic digestion and peptide extraction was followed by mass spectrometry. MALDI-TOF analysis of the unfractionated tryptic digest of the appropriate gel band identified 79% of the masses detected as predicted tryptic cleavage products of human HMGB1 (Figure 2). These peptides represented approximately 56% of the protein sequence (see bold letters). The identity of 4 peptides: Lys^30^-Lys^43^, His^31^-Lys^43^, Ile^113^-Lys^127^, and Tyr^155^-Arg^163^ was further confirmed by CID analyses. Masses, corresponding to predicted His-tag tryptic peptides, were also detected, their identity was confirmed by PSD analysis. Similarly, 82% of the masses detected in the second positive standard sample (i.e., recAtn) matched predicted tryptic peptides of the rat HMGB1. The identity of m/z 1520.76 as the predicted Ile^113^-Lys^127^ peptide was further confirmed by PSD analysis. These peptides represented approximately 47.6% of the protein sequence (Figure 3, see bold letters).

**HMGB1 ELISA**

For a more sensitive estimation of the HMGB1 inducing ability of *M. bovis* BCG and its comparison with other inducers, the HMGB1 concentrations were determined with a recently established [24] available ELISA kit (Shino-Test Corp, Shagamihara, Japan). At the same time, we wished to check whether the HMGB1 induction proceeded in parallel with the TNF-α induction (Table 1). LPS resulted in a moderate TNF-α production in U-937 cells (55 ± 6 pg/mL), and a weak HMGB1 secretion (84 ± 12 ng/mL). There was a higher concentration of TNF-α in the supernatant of U-937 cells incubated with heat-killed *S. aureus* (650 ± 40 pg/mL), with a considerable amount of HMGB1 in the supernatant of the same cultures (150 ± 14 ng/mL). Much greater amounts of TNF-α and HMGB1 were measured in the PMA-treated cell supernatants (350 ± 50 pg/mL and 365 ± 49 ng/mL, resp.). The BCG strain resulted in a more pronounced HMGB1 secretion than *S. aureus* did (450 ± 44 ng/mL), though the TNF-α inducing capacity was lower than that of *S.aureus* (120 ± 11 pg/mL). So mycobacteria induced almost the same magnitude of HMGB1 as that induced by PMA. PMA and BCG added at the same time resulted in the highest HMGB1 secretion (645 ± 125 ng/mL; Table 1).

### 3.3. HMGB1 relocalizes from nucleus to cytoplasm in cells activated with BCG

**Figure 4:**
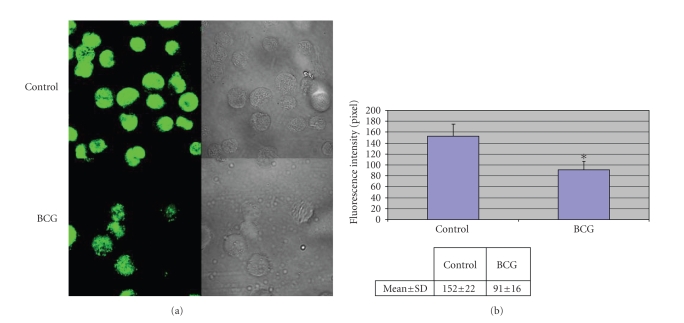
Immunofluorescence analysis of HMGB1 in U-937 cells. Cells were fixed, permeabilized, and stained with anti-HMGB1 chicken IgY, thereafter with secondary antibody (FITC-labeled anti chicken antibody), and analyzed with confocal microscopy.

The intracellular localization of HMGB1 in U-937 cells incubated with BCG was investigated by immunofluorescence and analyzed by confocal microscopy. Nonstimulated cells displayed strong staining for HMGB1 mostly restricted to the nucleus. Eighteen hours after stimulation with BCG, HMGB1 appeared to move from the nucleus, which is still partly positive to the periphery of the cells, where it displayed a punctuate staining in the cytoplasm. The fluorescence intensity therefore was significantly lower in BCG stimulated cells, because of the dispersity of the fluorescence (Figure 4).

## 4. DISCUSSION

The present study demonstrates that U-937 cell model is reliable and highly reproducible for determination of HMGB1 secretion and TNF-α production after infection with *M. bovis* BCG strain.

Although TNF itself can induce HMGB1 release, it contributed only partly in HMGB1 release in our experiments. The secretion of TNF-α from the U-937 cells was relatively high following incubation of the cells wih heat-killed *Staphylococcus aureus*, and there was considerable HMGB1 secretion. In our experiments *E. coli* LPS induction resulted in a lower TNF production and a weak HMGB1 secretion from the monocytic cells (Table 1). *Mycobacterium bovis* BCG resulted in a much lower TNF-α production, but it induced a considerable amount of HMGB1. We speculate that the receptors (i.e., Toll-like receptors) recognizing cell wall components of Gram-positive and Gram-negative bacteria may not be equally involved in HMGB1 secretion, which serve as HMGB1 receptors too [25]. Moreover, the intracellular mycobacteria may activate different signals which lead to HMGB1 secretion. TNF plays a critical role in host defense against *Mycobacterium tuberculosis* [26]. Mycobacteria are able to induce TNF production by different pathways [27]. However, it is very likely that this is not the only way to induce HMGB1 release. Further studies are necessary to elucidate the exact mechanism, that is, which signalization is initiated by *Mycobacterium bovis* resulting in HMGB1 secretion.

In our immunofluorescence experiments, the translocation of HMGB1 from the nucleus toward the cytoplasm was observed following induction of cells with BCG, which proves an active secretion process. The HMGB-1 protein can get out of the cells by two mechanisms: it can be secreted actively mostly by living inflammatory cells, or released passively by damaged or necrotic cells. HMGB-1 lacks a secretory signal peptide (similarly to interleukin-1β (IL-1β) [28]), so it can not be secreted via the Golgi-endoplasmic reticulum pathway. In resting monocytes, HMGB1 shuttles through the nuclear membrane to the cytosol or into the nucleus. The activation signal leads to the acetylation of lysine residues within the two nuclear localisation signal (NLS) sites on the HMGB-1 molecule, thereby neutralising their charge and making them unable to function as NLS. This is thought to inhibit the relocalisation of the protein into the nucleus, so the hyperacetylated HMGB-1 accumulates in the cytosol and will be packed into secretory lysosomes. Hence, we conclude that the translocation of HMGB1 from the nucleus toward the cytoplasm, which was observed in our immunofluorescence experiment following the induction of the cells with BCG, may lead to an active secretion process.

Though U-937 cells are able to release a low level of HMGB1 spontaneously, it is noteworthy, that they secrete a considerable amount of HMGB1 only following PMA activation (Figure 1 and  Table 1). PMA is a potent activator of monocytes, resulting in differentiation too. Thus, it is very probable that activation, or (much more likely) differentiation of monocytes is an important factor which may facilitate their HMGB1 secretion. Incubation of the cells with BCG resulted in HMGB1 secretion even without a coincubation with PMA. However, the cells treated with PMA and BCG simultaneously secreted the highest amount of HMGB1 (Figure 1 and Table 1).

The results of the ELISA measurements correlated well with those of the Western blot experiments, but the sensitivity was higher. No ultrafiltration was needed before testing of the cell supernatants, as it was necessary in Western blot experiments.

In the Western blot experiments the chicken anti-HMGB1 antibody recognized both the *E. coli*-derived recombinant human HMGB1, and the recombinant rat HMGB1, but the two proteins featured slightly different molecular weights by SDS-PAGE (30 kD versus 25 kD). In order to determine the structural differences, both proteins were in-gel digested with trypsin and the resulting peptide mixtures were subjected to MALDI-TOF mass and PSD analysis. These results confirmed the identity of the proteins. Our “natural” samples, from the cell supernatants gave positive bands with this antibody at the same molecular weight, as that of rat HMGB1. The exact reason for this is not yet known. It is very likely, that naturally secreted HMGB1 from biological sources is not identical to the *E. coli*-derived recombinant HMGB1, and they may be recognized differently by different antibodies. This might be the explanation that why not all commercial monoclonal antibodies are equally able to detect HMGB1 from biological samples in Western blot experiments (personal observations). The chicken anti-HMGB1 antibody was successfully applied also to detect human HMGB1 from sera of septic patients in the study of Sundén-Cullber et al. [20].

HMGB1 can occupy a central role in mediating the local and systemic responses to invasion by pathogens. Our pilot experiments draw attention to the HMGB1—inducing ability of *Mycobacterium bovis*, demonstrated in both Western blot and ELISA experiments. Assessment the pathophysiological role of this late cytokine in mycobacterial infections demands further in vitro and in vivo experiments.

## Figures and Tables

**Table 1:** HMGB1 and TNF-α production of U-937 cells.

	HMGB1 ng/mL	TNF-α pg/mL
Control	28 ± 5	15 ± 10
LPS 100 ng/mL	84 ± 12*	55 ± 6*
SA	150 ± 14*	650 ± 40*
BCG	450 ± 44*	120 ± 11*
PMA 5 ng/mL	365 ± 49*	350 ± 50*
PMA + SA	580 ± 15*	850 ± 120*
PMA + BCG	645 ± 125*	900 ± 150*

U-937 cells were induced for 24 h with LPS, heat-killed *Staphylococcus aureus* (SA), 5 ng/mL PMA, and *Mycobacterium bovis* BCG. Supernatants were processed for ELISA determination as it was described in Section 2. Data are means ± SD of the results of 5 experiments.

*P *<* .001 versus Control.
